# Retention Ability of a Glass Carbomer Pit and Fissure Sealant

**DOI:** 10.3390/ijerph19041966

**Published:** 2022-02-10

**Authors:** Liana Beresescu, Monika Kovacs, Alexandru Vlasa, Alexandra Mihaela Stoica, Csilla Benedek, Mihai Pop, Denisa Bungardean, Daniela Eșian

**Affiliations:** Faculty of Dental Medicine, University of Medicine and Pharmacy, Science and Technology George Emil Palade, 540139 Târgu-Mureș, Romania; liana.beresescu@umfst.ro (L.B.); monika.kovacs@umfst.ro (M.K.); csilla.bukhari@umfst.ro (C.B.); mihai.pop@umfst.ro (M.P.); denisa.bungardean@yahoo.com (D.B.); daniela.esian@umfst.ro (D.E.)

**Keywords:** dental sealer, glass carbomer sealer, composite resin sealer, dental decay

## Abstract

Dental sealants are an excellent means to prevent pits and fissure decay. Currently, there are multiple commercially available sealant materials. The purpose of this study was to assess the retention of glass carbomer fissure sealant and the incidence of secondary caries over a period of 24 months in comparison with a resin-based sealant. Materials and Methods: We included 32 children in the study, with ages between six and eight years and an average age of 6.8 years old. For each child, we sealed four permanent molars (totaling 128 teeth). The study group was divided into sub-groups. Sub-group A was represented by 64 first permanent molars which underwent dental sealing procedures with composite resin-based fissure sealant, Helioseal F™, and sub-group B was represented by 64 first permanent molars which underwent dental sealing procedures with glass carbomer cement, GCP Glass Seal™. The sealants were assessed clinically at 6, 12, 18, and 24 months. Results: The 6-month follow-up evaluation showed no statistically significant differences between the two materials neither regarding sealant retention nor new carious lesions formation (*p* > 0.05). At the 12-month recall, 57 molars had good retention (89.06%) from sub-group A and 44 molars (68.75%) from sub-group B; there was a statistically significant difference (*p* = 0.0187) between the two treatment choices only regarding material retention. At the last recall after 2 years, sub-group A had a higher number of molars with perfect sealing (47–73.43%) and 8 molars (12.5%) with new caries lesions and sub-group B had 23 (35.93%) molars with perfect sealing and 15 molars (23.44%) with new caries lesions; there was a statistically significant difference (*p* < 0.0001) between the two treatment choices only regarding material retention. Conclusions: The glass carbomer retention is very inferior to the resin-based material. The glass carbomer sealant was effective in preventing new caries development, comparable with the conventional resin-based sealant.

## 1. Introduction

The high prevalence of dental caries in the pits and fissures of molars is well known, and the suppression of these retentive spaces by the sealing method, to prevent the occurrence of decay lesions, has been widespread in recent decades [1]. This procedure proved to be effective in preventing dental caries, a fact that has been demonstrated over the years by several studies [2,3,4]. However, the efficiency of the sealing materials is limited, due in particular to poor retention. Their prophylactic effect is mainly attributed to their adhesion to the enamel surface and physical “filling” of the pits and fissures, which become isolated from the oral cavity environment [4]. As long as the sealant remains intact, caries will not develop under it [3]. Therefore, the retention of the sealing material is the main condition for successful sealing [5]. More recent studies, however, emphasize the role of another quality of the sealing materials that increases their efficiency, namely biological activity [4,5,6]. Nowadays, there are two types of pit and fissure sealants available, widely used in dental practice: resin-based and glass-ionomer-based materials [6]. Composite resin materials are currently the first choice for sealing because of their superior retention compared to glass ionomer cements, as documented by several recent studies [7,8]. Regarding the biological properties, composite resins are inert materials, except for the release of fluoride, which has been incorporated as of the fourth generation of composite resins [8]. On the other hand, glass ionomers have retention inferior to composite resins; nevertheless, they are biologically active. However, fluoride release and the potential to increase fluoride concentration in the enamel is lower in the case of composite resin usage compared to glass ionomer cements [9,10]. Glass ionomer sealing materials are made from a naturally bioactive material that chemically attaches to the tooth structure. This bond between the glass ionomer and tooth is strong and durable, and it is due to an ion-exchange process [11,12,13]. Glass ionomer cements release active ions which have multiple biological roles [14]: phosphate, sodium, and calcium ions occur in saliva and have an important role in buffering demineralization and supporting the remineralization of dental structure. Calcium is the major constituent of hydroxyapatite; silicate can also be incorporated with a beneficial effect in the hydroxyapatite structure [15,16]. Additionally, glass ionomers release fluoride, which can help the formation of fluorapatite at the tooth surface. This new structure is 10 times less soluble than hydroxyapatite [17]. Glass ionomers have the capability of taking up ions such as phosphate and calcium ions and form a more resistant surface [18]. They could develop a new “enamel-like” structure at the bottom of pits and fissures [19]. If the external fluoride concentration is high enough, glass ionomers can use it and gradually release it afterwards [20,21]. Currently, when adequate isolation [11] cannot be achieved, and the caries risk of the individual is very high [22,23] due to their inferior retention and reduced sensitivity to moisture compared to composite resins, they are used as a short-term sealing material in partially erupted teeth. Recently, a new material called glass carbomer was developed in order to achieve better long-term results. Glass carbomer cements are monomer-free, carbomized, nano-glass restorative cements developed from a conventional glass ionomer material, which contain nanoparticles of hydroxyapatite and fluorapatite [20]. Although previous reports suggested that the filler was fluorapatite [21,22], an NMR (nuclear magnetic resonance) spectroscopy analysis has shown that hydroxyapatite is actually the filler [23].

The advantages of glass carbomer, compared to traditional glass ionomer cements, are the following: improved chemical and mechanical properties [24,25] in terms of resistance, flexural strength, wear, remineralization power, and command setting—with an LED-curing device [24]. The heat generated using an LED-curing device has been shown to accelerate the setting process of glass ionomer cements [26], increase their compressive strength [27], and decrease microleakage formation [28]. In particular, the shear bond strength of glass carbomer was reported to be comparable or higher than conventional glass ionomer cement [29]. Additionally, there are some indications that these materials might be capable of transitioning into apatite-like and enamel-like materials [19]. Recent studies have reported that glass carbomers release fluoride at higher levels than conventional glass ionomers [30,31]. Regarding fluoride uptake behavior, the level noted in glass carbomer was also substantially higher than that in glass ionomer cements; even this increased fluoride uptake seems not to lead to a high fluoride release [32].

The aim of this study was to assess the retention ability and the incidence of secondary caries associated with a glass carbomer fissure sealant in comparison with a resin-based sealant over a period of 24 months.

## 2. Materials and Methods

The study methodology was approved by the ethics committee of the Denta Aur Private Medical Center, Targu-Mures, Romania. The clinical evaluations and procedures were performed during the period of May 2018 until May 2020. Before examination, the parents of all participating children gave written informed consent. All the patients were instructed to have appropriate oral hygiene and diet. The recommended technique was Bass, being accepted by all patients and also being very effective in removing dental plaque. They were trained and motivated to brush their teeth using soft plastic toothbrushes. Regarding diet, children were advised to limit sugar intake and to eat sweets only after meals, not between them.

Two calibrated operators performed all the clinical steps, helped by a dental assistant. Before the first examination, dentists participated in an ICDAS-II calibration course, evaluating the condition of tooth surfaces and the presence of caries according to the International Caries Detection and Assessment System (ICDAS-II), and they also had the opportunity to familiarize themselves with the sealant’s application technique.

Sample size determination. The sample size required was determined to be 128 teeth (64 teeth per group) using G-power software™ Heinrich Heine University, Dusseldorf, Germany, for Windows, for a power of 95% (α = 0.05, 1 − β = 0.05).

Initially, a total of 47 children were included in the study. The selection of children was made based on the school grade of first class which corresponds to the age of 6 to 8 years old, with an average age of 6.8 years old.

For the clinical examination of molars, in order to check the indication of pit and fissure sealing, we used two examination methods: visual and tactile. For the visual examination, we cleaned and dried the teeth, and for the tactile examination, we used a rounded-tip dental probe to clean the plaque and food debris from the pits and fissures system [4]. If the integrity of the occlusal tooth structure was doubtful, the suspicious teeth/children were excluded from the study.

The inclusion criteria were as follows:

- Permanent molars completely erupted with deep pits and fissures susceptible to tooth decay.
- No clinical signs of the dental caries process.
- Teeth without dental abnormalities.

The exclusion criteria were as follows:

- Partially erupted permanent molars.
- Clinical signs of dental caries.
- Teeth with fillings or sealants.

Out of 47 children, 32 had all 4 of their permanent first molars with an indication for sealing. In total, 32 children continued to participate in our study. We chose to use the split-mouth design method [33]. Therefore, 128 teeth from the 32 children were included in the study.

The molars were divided into 2 sub-groups depending on the applied sealant material:

- Sub-group A:—64 molars—Helioseal F™, Ivoclar Vivadent Schaan, Liechtenstein—conventional resin-based sealant;
- Sub-group B—64 molars—GCP Glass Seal™, GCP Dental b.v. Mijlweg the Netherlands—glass carbomer sealant group.

Sealant application steps were performed according to the manufacturer’s instructions strictly following each working step.

For conventional resin-based sealant, the steps were: professional dental cleaning, rinsing with water and air-drying, meticulous isolation of the tooth, air-drying of the occlusal surface, enamel etching with 37% phosphoric acid gel for 30 s, rinsing with water and air-drying the tooth, control of the acid-etched dental surface, sealant application on the conditioned enamel surface, light curing of the sealant, control of marginal adaptation, and occlusion control.

For the glass carbomer sealant group, after professional dental cleaning, the enamel was conditioned with a conditioner (EDTA solution) for 20 s, rinsed, dried but not desiccated, and kept isolated with cotton-rolls; the glass carbomer material was applied, then GCP gloss was spread with a pellet onto the sealed surface and was light-cured for 60 s with a polymerization unit. After marginal adaptation and occlusion control, the patient was instructed not to eat for half an hour.

Regular post-placement evaluations were performed at intervals of 6 months over a period of 24 months. At every check-up, integrity and marginal adaptation of sealant materials were assessed through visual and tactile examination.

Sealers were assessed according to Simonsen’s criteria [34]:

Group I: completely retained.

Group II: partially retained.

Group III: missing.

### Statistical Analysis

For the evaluation of the categorical data, we used the chi square test. The chosen significance level was set at 0.05, and *p* was considered significant when *p* ≤ 0.05. All data were recorded using GraphPad Prism™ V6.01 software for Windows™ 2017.

## 3. Results

The 6-month follow-up evaluation showed no significant differences between the two sealing methods, neither concerning sealant retention (*p* > 0.05) nor new carious lesions formation (Table 1 and Table 2).

The 12-, 18-, and 24-month follow-up intervals (Table 1 and Table 2) showed significant differences between the two treatment choices only regarding sealant retention (*p* < 0.05). The resin-based sealant material (Helioseal) retention rates were higher than those of the glass carbomer (GCP glass seal) rates. There was no significant difference regarding the incidence of new carious lesions (*p* > 0.05) between the two materials we used.

## 4. Discussion

The children included in our study were 6 to 8 years old, which is ideal for the sealing of the first permanent molar, since its eruption usually starts at 6, while its root development continues post-eruption until the age of 10 [35]. During this time interval, caries receptivity is at its maximum.

The split-mouth design [33] is frequently used in dental clinical research [36] because it has the advantage of removing a lot of inter-subject variability. Every subject received sealants on all four molars, with no bias when placing the sealant. This method ensures that we had equal numbers of sealed molars in the upper and lower jaws and in the right and the left sides of each studied material.

Our findings are similar to some previously published investigations [37,38,39] revealing a superior retention of the composite resins compared to the glass carbomers.

The number of carious lesions in the group of teeth sealed with glass carbomer was higher than in the group of teeth sealed with composite resin at 12-, 18-, and 24-month recall; the difference between the incidence of carious lesions in the two groups increased as the observation period increased, but there was not any statistically significant difference, regardless of the recall intervals. The retention of the glass carbomer, however, was much lower than that of composite. This was probably due to the caries-preventive and enamel-remineralization properties of the glass carbomer.

Other studies also suggest that even in the early exposure of enamel to the oral environment as a result of the loss of the sealant, there is no difference between caries rates in teeth sealed with glass-ionomer-based sealants and resin-based sealants [39,40]. According to some authors [41], the retention of the glass-ionomer-based sealing material has a secondary importance; the real objective is the prevention of the occurrence of new carious lesions. The risk of retention loss of the sealant is only associated with the risk of new carious lesions in resin-based sealant; for glass-ionomer-based sealants, retention is not a valid predictor [42]. Other studies also suggest that the mere presence of material remnants in the fissures after the loss of the sealant can still ensure caries prevention [43,44]. Related to this, some studies noticed that glass ionomer materials form a new structure in the pits and fissure depth. This new structure has a better resistance than the pits and fissure original structure because it has an increased content of minerals (especially calcium and phosphate) [37].

Under the conditions of our survey, the two materials tested showed similar efficiency in preventing caries. Based on our data, we could not associate plaque indexes and sugar intake (or other factors that might affect caries development) with the incidence of new caries lesions. All children had good oral hygiene and their parents strived to motivate them in achieving this goal. Regarding the manipulation of these two materials, glass carbomers have the advantage of being easier to handle and less time-consuming because etching with acid is not necessary. This fact was also noticed in other, similar studies [38,45].

### Limitations of the Study

The results of this study have limitations, because of the small-numbered study group, the specific population (school children in Targu-Mures), and due to the short observation period.

## 5. Conclusions

Glass carbomer fissure sealants display a lower retention rate than composite resin fissure sealants. They are similarly efficient in preventing caries development. However, longer follow-up studies are needed in order to obtain more comprehensive data.

## Figures and Tables

**Table 1:** Sealant retention rate after 6, 12, 18, and 24 months according to the evaluation criteria of Simonsen.

Sealant retention after 6 months					
	I	II	III	Total	
GCP glass seal	60 (93.75%)	0	4 (4.69%)	64	
Helioseal	64 (100%)	0	0	64	*p* = 0.1191
Total	124 (96.88%)	0	4 (4.69%)	128 (100%)	
Sealant retention after 12 months.					
	I	II	III	Total	
GCP glass seal	44 (68.75%)	5 (7.81%)	15 (23.44%)	64	*p* = 0.0187
Helioseal	57 (89.06%)	2 (3.13%)	5 (7.81%)	64	
Total	101 (78.91)	7 (5.47%)	20 (15.63%)	128	
Sealant retention after 18 months.					
	I	II	III	Total	
GCP glass seal	37 (57.81%)	6 (9.38%)	21 (32.81%)	64	*p* = 0.0253
Helioseal	51 (79.68%)	2 (3.13%)	11 (17.19%)	64	
Total	88 (68.75%)	7 (5.47%)	32 (25.00%)	128	
Sealant retention after 24 months.					
	I	II	III	Total	
GCP glass seal	23 (35.93%)	3 (4.69%)	38 (59.38%)	64	*p* < 0.0001
Helioseal	47 (73.43%)	5 (7.81%)	12 (18.75%)	64	
Total	70 (54.69%)	8 (6.25%)	50 (39.06%)	128	

**Table 2:** Incidence of new carious lesions after 6, 12, 18, and 24 months.

New carious lesions after 6 months				
	Yes	No	Total	
GCP glass seal	0	64	64	
Helioseal	0	64	64	
Total	0	128	128	
New carious lesions after 12 months				
	Yes	No	Total	
GCP glass seal	4 (6.25%)	60 (93.75%)	64	*p* = 1.000
Helioseal	3 (4.68)	61 (95.31)	64	
Total	7 (5.47)	121 (94.53%)	128	
New carious lesions after 18 months				
	Yes	No	Total	
GCP glass seal	6 (9.37%)	58 (90.63%)	64	*p* = 1.0000
Helioseal	5 (7.81%)	59 (92.19%)	64	
Total	11 (8.59%)	117 (91.41%)	128	
New carious lesions after 24 months				
	Yes	No	Total	
GCP glass seal	15 (23.44%)	49 (76.56%)	64	*p* = 0.1663
Helioseal	8 (12.5%)	56 (87.50%)	64	
Total	23 (17.97%)	105 (82.03%)	128	

## Data Availability

All data regarding this manuscript can be checked with corresponding authors.
